# Rift Valley fever seroprevalence in ruminants in Dhobley town, Lower Juba region, Somalia, in 2021

**DOI:** 10.1017/S0950268825100599

**Published:** 2025-10-10

**Authors:** Ahmed A. Hassan-Kadle, Aamir Muse Osman, Abdulkarim A. Yusuf, Mohamed A. Shair, Osman A. Hassan, Rachel Maluleke, Antoinette Van Schalkwyk, Marco Romito, Alison Lubisi, Abdalla M. Ibrahim, Rafael F.C. Vieira

**Affiliations:** 1 Somali One Health Centre, Abrar University, Mogadishu, Somalia; 2 Abrar Research and Training Centre, Abrar University, Mogadishu, Somalia; 3 Graduate Program on Veterinary Sciences, Universidade Federal do Paraná, Curitiba, Brazil; 4 Department of Animal Health and Veterinary Service, Ministry of Livestock, Forestry, and Range, Mogadishu, Somalia; 5 Department of Slaughterhouse, Somali Meat Company, Mogadishu, Somalia; 6 Department of Animal Health and Veterinary Service, Ministry of Livestock, Forestry, and Range, Jubaland State, Somalia; 7 Agricultural Research Council, Onderstepoort Veterinary Research, Pretoria, South Africa; 8 Department of Epidemiology and Community Health, The University of North Carolina at Charlotte, Charlotte, NC, USA

**Keywords:** Rift Valley Fever, Seroprevalence, Ruminants, One Health, East Africa

## Abstract

This study assesses the seroprevalence of Rift Valley fever (RVF) in ruminants in Dhobley, Somalia, following a 2021 outbreak in Kenya. Among 142 ruminants sampled, 4.9% were seropositive for RVF virus (RVFV) antibody, with IgM antibodies (1.4%) indicating recent exposure, though no cases were RT-PCR-positive. Unregulated livestock movement and limited surveillance pose significant risks for future outbreaks, underscoring the need for enhanced surveillance systems and One Health strategies.

## Introduction

Rift Valley fever (RVF) is a mosquito-borne disease caused by the Rift Valley fever virus (RVFV), which belongs to the genus *Phlebovirus* and the family *Phenuiviridae* [1, 2]. The disease is endemic to Africa and is characterized by abortions and neonatal deaths in domestic ruminants and a range of human illnesses, from flu-like symptoms to encephalitic, ocular, or haemorrhagic syndromes [1, 2]. RVF poses a major global threat to both human and animal health. The widespread distribution of competent mosquito vectors, along with increased international travel and livestock trade, has facilitated the spread of RVFV throughout much of Africa, including Somalia and the Middle East [2, 3]. This situation underscores the growing concerns about RVFV’s pandemic potential [4], leading the World Health Organization (WHO) to include RVF in its Blueprint priority diseases list for research and development [5].

Somalia, a country that is heavily reliant on livestock and shares porous borders with Kenya and Ethiopia, where zoonotic transboundary diseases are frequently reported [6, 7], lacks systematic vaccination programmes and comprehensive surveillance for RVFV. Limited access to laboratory infrastructure and resources leads to underreporting of outbreaks, posing significant challenges to timely detection and control. Additionally, the unregulated cross-border movement of livestock between Somalia and neighbouring countries such as Kenya, which experienced an RVF outbreak in 2021 [8], increases the risk of spillover infections in both animal and human populations in Somalia. Previous serological studies documented an RVF antibody prevalence of 1% in cattle, 5% in goats, and 2% in sheep in Somalia [3]. However, the last confirmed RVF outbreak occurred in 2007 in the southern regions of Middle Juba, Lower Juba, and Gedo, bordering Kenya, where the focal point of the outbreak was identified [9].

The recent declaration of an RVF outbreak in neighbouring Kenya in 2021 underscores the urgent need for enhanced monitoring and preventative strategies. In response to the WHO’s February 2021 announcement identifying outbreaks in Kenyan districts bordering Somalia, Abrar University, in collaboration with Somalia’s Federal and State Ministry of Livestock, Forestry, and Range, conducted an investigation in Dhobley, Jubaland State, Somalia, in April 2021 to assess the circulation of RVFV in the country.

## The study

A cross-sectional study was conducted in April 2021 in Dhobley Town (0°24′38″N, 41°0′35″E), Jubaland State, Somalia. Samples were collected from 18 herds using a non-probabilistic convenience sampling method. A total of 142 ruminant blood and serum samples were obtained, comprising 100 adults and 42 young animals, with 123 females and 19 males. The sampled animals included 54 goats, 54 sheep, and 34 cattle. The study area is located near the Kenyan border, where an RVF outbreak had been declared by the Kenyan government and the WHO in February 2021 [8]. The sampled animals were managed under an extensive husbandry system, with frequent cross-border grazing. No clinical signs suggestive of RVF were recorded during sampling.

Sera were tested for the presence of anti-RVFV antibodies and IgM-specific immunoglobulins using ID Screen® Rift Valley Fever Competition Multi-species and ID Screen® Rift Valley Fever IgM Capture ELISAs, respectively (Innovative Diagnostics, Grabels, France). IgM-positive samples were further tested via RT-PCR [10] at Onderstepoort Veterinary Research, South Africa, to confirm recent infection. Data were compiled and analysed using Epi Info™ software, version 7.2.3.1 (Centers for Disease Control and Prevention, CDC, USA).

Of the 142 ruminants, seven (4.9%, 95% CI: 2.0–9.9) tested seropositive for RVFV, and all seropositive animals were female. Anti-RVFV IgG seroprevalence was 4.2% (6/142), while anti-RVFV IgM prevalence was 1.4% (2/142). None of the IgM-positive samples tested positive by RT-PCR. Goats exhibited the highest RVFV seroprevalence, with 4/54 (7.4%, all adults) testing positive for anti-RVFV IgG, including one goat positive for both IgG and IgM. Among cattle, 2/34 (5.9%, both young animals) tested positive for anti-RVFV IgG, and 1/34 (2.9%) for anti-RVFV IgM. All sheep samples tested negative for both anti-RVFV IgG and IgM antibodies.

## Conclusion

This study reveals a concerning prevalence of RVF in ruminants from Dhobley Town, Somalia. Although no cases were confirmed by RT-PCR, which may have been caused by the short viraemic period, the detection of anti-RVFV IgM antibodies indicates recent exposure to RVFV. The close proximity of Dhobley Town to the Kenyan border, where a recent RVF outbreak was reported, raises concerns about silent RVFV circulation. Unregulated cross-border livestock movement facilitates the spread of diseases across borders, increasing the risk of spillover infections.

Although the observed seroprevalence was low, it is crucial to recognize that these findings likely represent an underestimation of the true epidemiological situation due to the acknowledged underreporting and the absence of routine RVF surveillance systems in Somalia. A key limitation of this study is the lack of recorded clinical data, including abortion history or observable signs of illness, since such information was not collected at the time of sampling. This gap restricts the ability to correlate serological findings with clinical outcomes, which would have added value to the epidemiological interpretation.

These findings raise concerns about the possible silent circulation of RVFV in the region, which could lead to future outbreaks under favourable ecological conditions. The lack of recent outbreak data, coupled with limited laboratory capacity, indicates that the true burden of the disease in Somalia remains largely unknown, hindering effective preparedness and response efforts. Therefore, this study highlights the urgent need for the establishment and strengthening of a surveillance system in Somalia. This includes enhancing diagnostic capacity at the laboratory level and implementing a coordinated One Health approach that integrates animal and human health monitoring. Such measures are essential to accurately detect, effectively prevent, and rapidly control localized RVF outbreaks, thereby reducing the significant risk of escalation into a broader epidemic with detrimental impacts on both livestock livelihoods and public health security in the region.

## Data Availability

All data generated or analysed during this study are included in this article.
